# Protein-DNA docking with a coarse-grained force field

**DOI:** 10.1186/1471-2105-13-228

**Published:** 2012-09-11

**Authors:** Piotr Setny, Ranjit Prasad Bahadur, Martin Zacharias

**Affiliations:** 1 Physics Department T38, Technical University Munich, James Franck Str. 1, 85748 Garching, Germany; 2 Indian Institute of Technology Kharagpur, Kharagpur - 721302, India

## Abstract

**Background:**

Protein-DNA interactions are important for many cellular processes, however structural knowledge for a large fraction of known and putative complexes is still lacking. Computational docking methods aim at the prediction of complex architecture given detailed structures of its constituents. They are becoming an increasingly important tool in the field of macromolecular assemblies, complementing particularly demanding protein-nucleic acids X ray crystallography and providing means for the refinement and integration of low resolution data coming from rapidly advancing methods such as cryoelectron microscopy.

**Results:**

We present a new coarse-grained force field suitable for protein-DNA docking. The force field is an extension of previously developed parameter sets for protein-RNA and protein-protein interactions. The docking is based on potential energy minimization in translational and orientational degrees of freedom of the binding partners. It allows for fast and efficient systematic search for native-like complex geometry without any prior knowledge regarding binding site location.

**Conclusions:**

We find that the force field gives very good results for bound docking. The quality of predictions in the case of unbound docking varies, depending on the level of structural deviation from bound geometries. We analyze the role of specific protein-DNA interactions on force field performance, both with respect to complex structure prediction, and the reproduction of experimental binding affinities. We find that such direct, specific interactions only partially contribute to protein-DNA recognition, indicating an important role of shape complementarity and sequence-dependent DNA internal energy, in line with the concept of indirect protein-DNA readout mechanism.

## Background

Protein-DNA interactions regulate many cellular processes involving gene expression, DNA replication and repair. The recognition process can either be specific or non-specific depending on functional requirements [1-6]. Although several structural studies have been performed to understand the specificity of the recognition process, the mechanism is still elusive and a simple code for DNA recognition by proteins does not seem to exist [7-10]. Despite the active interest in the field of experimental determination of the atomic structure of protein-DNA complexes in the last few decades, protein-DNA complexes represent only 3% of all the macromolecular structures submitted in the Protein Data Bank (PDB) [11].

For a large fraction of known and putative interactions structural knowledge is still lacking. In addition, gene regulatory processes often involve weak and transient protein-DNA interactions for which experimental structure determination using X-ray crystallography can be difficult or impossible. It is desirable to be eventually able to answer the question if a protein may interact with DNA and how, without performing the experimental structure determination.

Computational docking methods can provide structural models of complexes in cases where it is difficult or impossible to obtain an experimental complex structure. It is a predictive method based on the structures of the individual partners. Several methods for protein-protein docking have been developed and used extensively for the prediction of protein-protein complexes [12,13]. Their performance is frequently assessed in the Critical Assessment of PRedicted Interactions (CAPRI) [14] challenge. However, much fewer methods for systematic docking to predict the structure of protein-DNA complexes have been published so far. This includes Fast Fourier correlation techniques that detect mainly shape complementarity and are widely used in the protein-protein docking field [15]. The geometric hashing method to detect locally matching surfaces has also been recently expanded for the prediction of protein-DNA complexes [16]. In addition, methods that include conformational changes at some stage of a multi-start docking search (program HADDOCK) have been applied to tackle the prediction of protein-DNA complexes [17,18]. The latter method requires as input additional data on the putative interaction regions of the partners [17], which is included as restraints during the docking search. The same authors have also developed benchmark sets for protein-DNA docking including partner structures in bound or unbound conformations [18].

One major challenge to any docking algorithm is to handle the size of the macromolecules, as the computational time for evaluating all relevant binding geometries increases rapidly with the number of involved atoms. The computational demand can be reduced by the use of a simplified representation of the macromolecules. In such approach, biomolecules are represented by pseudo atoms that represent geometric centers of complete chemical groups instead of single atoms. Such approach has already been successfully used for the analysis and prediction of biomolecule complexes [19-21].

One possibility to obtain the necessary interaction parameters is to relate them to the propensities of the considered pseudo atoms to stick together extracted from experimentally known structures. Gathering statistics for all possible pairs of the introduced atom types and using Boltzmann inversion to convert the probability of their finding together to (pseudo) energy functions, one can construct so called knowledge-based force field. In principle, such force field can not provide a valid representation of the free energy [22]: the structures gathered in Protein Databank (PDB) do not constitute an ensemble in the sense of statistical mechanics, the considered probabilities, and hence (pseudo) energies depend on an arbitrary definition of a reference state, and the thermodynamics of complex formation can not be dissected into pairwise additive terms. On the other hand, however, knowledge-based potentials are useful due to their simplicity. They implicitly incorporate many otherwise difficult to quantify effects such as solvent mediated interactions, shifts in protonation states, or the contributions of local conformational entropy (actually including any explicit, physical terms to knowledge based potential is not straightforward, as a) to some, unknown, extent they will double implicitly present contributions, b) it is no clear what energy scale to use). In contrast, physically based atomistic approaches either require enormous computational efforts to obtain the necessary sampling or quickly loose their fidelity when merged with approximate methods. As an effect, in the context of macromolecular complexes they do not provide better overall accuracy than current knowledge based approaches [23].

In the present study we have developed a distance-dependent, knowledge-based coarse grained force field for evaluating protein-DNA interactions during docking. The scoring function is compatible with the coarse-grained models for proteins and RNA, implemented in the ATTRACT docking program [19,21], and is parametrized based on 119 non-redundant structures of protein-DNA complexes derived from the PDB. The force field was extensively tested in systematic docking searches on bound and unbound protein and DNA partner structures. The applicability for employing the approach to evaluate the sequence specificity and the effect of protein mutations on protein-DNA binding was also investigated.

## Methods

### Force field parametrization

The force field is an extension of previously parametrized knowledge based potential for protein-RNA docking [21]. The interaction model is based on distance-dependent Lennard-Jones type potential, with an additional form allowing for repulsive interactions:

(1) $$\;U_{\text{ij}}^{\text{attr}}(r)=\varepsilon_{\text{ij}}\left(\frac{\sigma_{\text{ij}}^{8}}{r^{8}}-\frac{\sigma_{\text{ij}}^{6}}{r^{6}}\right),$$

(2) $$\begin{array}{ll} & U_{\text{ij}}^{\text{rep}}(r)=\left\{\begin{array}{cc} U_{\text{ij}}^{\text{attr}}(r)+2U_{\text{ij}}^{m} & \text{for}\;r\leq r_{\text{ij}}^{m}\\ -U_{\text{ij}}^{\text{attr}}(r) & \text{for}\;r>r_{\text{ij}}^{m} \end{array}\right)\; \end{array}$$

Pairwise specific parameters *σ*_*ij *_and *ε*_*ij *_govern bead-bead interaction range and strength respectively. The above potentials are assumed to account for all effective protein – nucleic acid interactions, hence no additional electrostatic terms are introduced.

**Figure 1:**
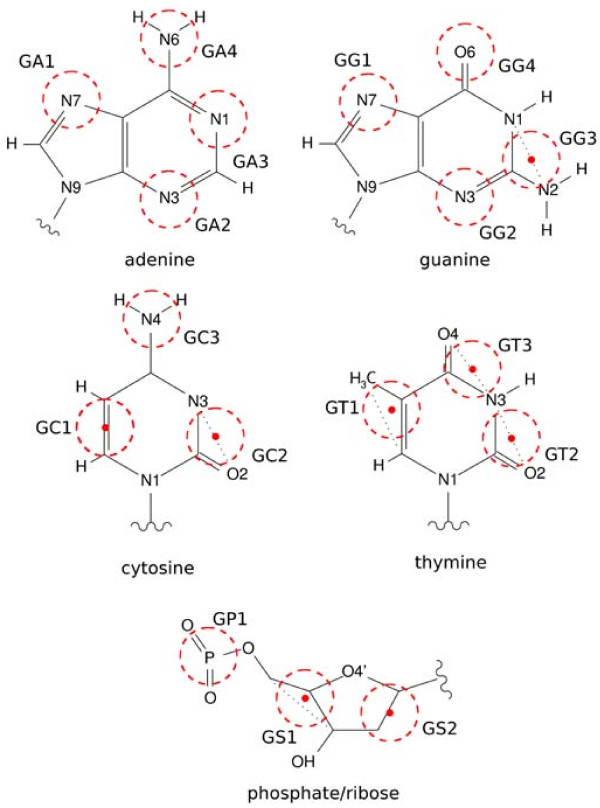
**Coarse-grained representation for DNA nucleotides.** Beads (dashed circles) are either placed on particular atoms or at geometric centers of a few atoms (denoted as dots).

The coarse-graining scheme (Figure 1) and interaction parameters for all DNA components shared with RNA remained intact. New bead types and parameters were introduced for thymine base (3 beads) and a part of ribose ring, containing C2’ carbon atom (GS2 bead).

In line with protein-RNA force field development scheme, the new parameters were obtained using a two stage procedure. First, starting from the existing values of their RNA counterparts (uracil beads in case of thymine, and ribose GS2 bead for deoxyribose GS2), *σ *parameters were optimized to provide possibly low root mean square deviation (RMSD) of protein C*α* atoms after rigid body energy minimization of native complexes. The optimization was performed using Monte Carlo-like approach in which trial parameter changes were accepted or rejected based on changes in RMSD score. Such score increasingly favored minimized solutions with low RMSD, but deemed unimportant RMSD variations, if RMSD was below 1 Å. The optimization procedure was repeated until no further change was observed among the optimized *σ *values.

The second optimization stage involved adjustments of *ε *parameters, and was performed in a similar manner. Here, the goal was to enhance scoring of native-like complexes with respect to generated decoys (200 for each training set structure). The acceptance of new *ε* values was driving towards the maximum number of properly ranked (i.e. with rank 1) native-like complexes, and maximum score favoring possibly high ranks of the remaining, non-optimal cases. After every 10 cycles of trial changes covering each *ε *value, a systematic docking search was performed for the test set structures. *ε *optimization was stopped after no further improvement in docking results was observed.

In order to disentangle the role of specific bead-bead interactions from the effect of shape complementarity in docking experiments, a non-specific force field variant was also prepared. The values of all *ε *parameters, governing the interaction strength between two given pseudoatoms, were equalized to an average *ε* value from the full protein-DNA force field. At the same time, *σ *parameters, governing the interaction range, were left unchanged.

### Data sets

The PDB was scanned for entries representing protein-DNA complexes. About 588 entries reporting X-ray structures at resolution 3.0 Å or better and including both a polypeptide chain of 50 or more amino acid residues and a polyribonucleotide of 5 or more nucleotides were found. In order to remove redundancy, when the protein components in two entries had more than 30% identity, we kept the one having better resolution and more structural completeness for further analysis. We have also visually checked each structure with molecular visualization software, and when there were two identical molecules in one asymmetric unit we kept only the one.

The final list of 117 complexes is reported in Additional file 1: (Table S1), and is used for the development of the force filed. The set was randomly divided into training (88 complexes) and test (29 complexes) sets.

### Docking protocols

All docking simulations were carried out with the ATTRACT program [19]. Both binding partners: receptor (DNA) and ligand (protein) were considered in coarse grained representation, and were treated as rigid bodies. Docking runs were based on the minimization of ligand potential energy in translational and rotational degrees of freedom, in the field of a fixed receptor. No additional information regarding distance constraints nor the location of binding region was used.

Systematic docking search was performed as series of energy minimization runs, starting from positions evenly distributed around the receptor at distances precluding receptor-ligand overlaps. For each such position, a set of multiple initial ligand orientations was considered. For the results presented herein, a spacing of ∼12 Å between starting points, and 208 orientations per starting point were used, providing a reasonable compromise between sampling accuracy and computational time. Each energy minimization was divided into 5 stages, with distance cutoff for pairwise interactions decreased in a stepwise manner from 50 to 8 Å.

Converged solutions were clustered according to their pairwise RMSD, and scored according to their potential energy. The evaluation of docking results quality was based on interface RMSD (iRMSD) between the assembled and native complex, and the fraction of reproduced native contacts (*f*_*NC*_). The native interface was defined as consisting of protein and DNA beads found within 8 Å to each other in the crystal structure. A docked geometry was considered as a “hit”, with iRMSD ≤ 2 Å and *f*_*NC *_≥ 0.3, or iRMSD ≥ 1 Å with *f*_*NC *_≥ 0.5. Such criteria are equivalent to “hit” being “high” *or* “medium” quality solution according to the CAPRI challenge [24] guidelines. Separately, the statistics for hits of only ”high” quality (i.e. with iRMSD ≤ 1.0Å and *f*_*NC *_≥ 0.5) was determined.

### Experimental data

#### Binding free energies

Experimentally derived protein-DNA affinities, used for comparison with calculated binding energies, were taken from ProNIT database [25]. In order to gather thermodynamic data corresponding to comparable experimental conditions, database records were filtered to contain only measurements carried out in temperature ranging from 20 to 25°C, pH from 6.0 to 8.5, and ionic concentration below 200 mM. For each such database record, that matched an available crystallographic structure, an identity level between the provided DNA sequence and the sequence actually represented in PDB file was calculated. A required identity level for overlapping sequence parts was assumed to be at least 0.8 for each DNA strand. Finally, as the total length of DNA double strand may critically affect binding affinity, even if only part of it makes direct contacts with protein, a Tanimoto similarity coefficient, *t*_*c *_=* N*_*id*_/(*N*_1_ + *N*_2 _−*N*_*id*_) (where *N*_*id*_ is a total number of identical nucleotides, *N*_1 _and *N*_2_ are lengths of compared sequences), was calculated between experimental and crystallographic sequences, and cases with similarity lower than 0.5 were excluded.

Such procedure resulted in 15 crystallographic protein-DNA complexes with matching experimental binding free energies [26-43] (Table 1). If more than one measurement was available for a given structure, an average *ΔG* was calculated.

**Table 1:** Crystallographic protein-DNA complexes (designated by PDB id) with matching experimental binding free energies extracted from ProNIT database

**PDB**	**Chains**	***ΔG*/kcal mol**^**−1**^
1AAY	A/BC	−11.5 [26]
1AZ0	AB/CD	−17.4 [27]
1B72	AB/DE	−9.0 [28]
1BHM	AB/CD	−11.7 [29]
1CEZ	A/TN	−10.8 [30]
1CMA	AB/CD	−5.4 [31]
1D02	AB/CD	−8.1 [32]
1ECR	A/BC	−15.6 [33]
1IHF	AB/CD	−10.3 [34,35]
1PUE	E/AB	−9.7 [36]
1QRV	A/CD	−7.4 [37,38]
1YSA	CD/AB	−9.5 [39]
1PUF	A/DE	−9.2 [28]
1TRO	AC/IJ	−12.6 [40-42]
1UBD	C/AB	−8.5 [43]

A set including crystallographic structures of protein-DNA complexes with corresponding binding affinities provided by Zhang *et al.*[44] was considered as an alternative source of data. After removing structures that were determined by NMR, included modified bases or uracil, or contained only a single DNA chain, still the adopted selection criteria (especially those referring to sequence similarity) were not met in many cases. Nonetheless, the results for this set are provided to enable comparison with other studies.

#### Free energy differences

Thermodynamic data describing differences in binding affinities for sets of DNA mutants targeting their specific protein partners was taken from the work of Morozov *et al.*[45]. Two of the structures (6CRO and 1CKQ) were removed from the data set because of missing residues, and other two (1PUE and 1BHM) were not considered in calculations because binding specificity in their case is achieved predominantly owing to indirect readout mechanism [45].

All complexes considered for comparison with experimental data were prepared in the same way as structures used for training and testing. DNA mutants were constructed by isosteric replacement of the relevant base pairs, with no further refinement of DNA backbone geometry.

## Results and discussion

### Parametrization

Parametrization process started with the adjustments of *σ*parameters. After three rounds of optimization, during which trial increments and decrements were examined for each considered parameter, no more improvement in the adopted RMSD score was observed.

During the second parametrization stage *ε*parameters were adjusted. After 10 optimization rounds, each including 10 trial changes for each considered parameter, new values were still being accepted. In order to avoid overfitting, systematic docking searches were performed for both training and test sets after each optimization round. Force field scoring performance was evaluated for each set by considering the percentage of hits found within *n* lowest energy solutions and calculating an area under the resulting curve (AUC) for *n *∈ {1*.*100}. The AUC of 1.0 would be equivalent to all native-like solutions having the best rank (the lowest energy) among their corresponding decoys, and AUC of 0.0 would mean no hits at all within 100 lowest energy solutions.

**Figure 2:**
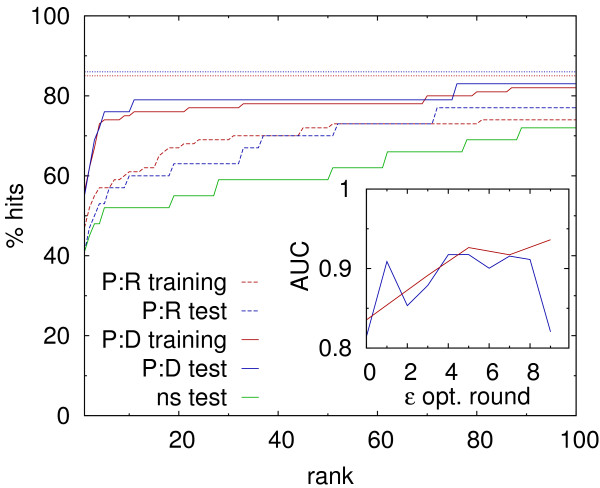
**Fractions of “hits” among 100 best ranking docked geometries for the original protein-RNA parameter set (P:R), optimized protein-DNA parameter set (P:D), and non-specific protein-DNA parameter set (ns).** Dotted lines show the respective fractions for all obtained geometries for P:D parameters. Inset: changes in AUC (see text for description) for the training set (red) and the test set (blue) during *ε*parameters optimization.

As expected based on the continuous acceptance of trial *ε*parameter changes, AUC for the training set was still increasing after 10 optimization rounds (Figure 2, inset, red line). At the same time, however, AUC for the test set (blue line) reached a plateau and started to drop, indicating a likely overfitting problem. Thus, the parameter set obtained after round 5 was selected as optimal and used in the following. The efficacy of newly obtained parameter set was evaluated based on its ability to provide stable energy minima consistent with experimental binding modes, and on its performance in systematic docking search. During potential energy minimization of crystallographic complexes, all structures remained stable in the sense that the converged energy minimum met the adopted criteria for a ”hit”. Furthermore, most (90%) of the optimized geometries in both training and test sets corresponded to high-quality solutions (Table 2). 

**Table 2:** Docking results – percent of hits and high quality solutions (in parentheses) – for protein-RNA (p-R), optimized protein-DNA (p-D) and non-specific protein-DNA (n.s.) parameter sets

	**Native**	**Top**	**Top**	**All**
**Set**	**minimized**	**scored**	**ten**	**docked**
				
p-R	100(90)	47(42)	61(53)	84(70)
p-D	100(92)	55(51)	75(64)	86(70)
n.s.	98(86)	34(31)	44(39)	73(57)
				
p-R	100(87)	41(41)	60(55)	90(62)
p-D	100(97)	55(55)	76(76)	86(86)
n.s.	80(47)	41(38)	52(41)	86(69)

Systematic docking search resulted in 55% of best ranked hits, and 75% of hits found within 10 best scoring solutions (Table 2). Again, no significant difference was found between training and test sets (Figure 2). When compared to the performance of original protein-RNA force field on the same structures, it is apparent that the parameter optimization process mainly improved scoring efficacy (increased the number of preferably ranked hits), while not affected the ability to find native-like geometries (did not change the number of generally found hits).

This can be understood, as probably the most important difference between RNA and DNA, contributing to specific protein-DNA recognition, is the additional methyl group of thymine. Its main effect relies on providing a unique hydrophobic interaction patch within the major groove of DNA, rather than on affecting local shape complementarity. Indeed, the comparison of optimized parameters for thymine with their uracil counterparts indicates significant increase (more than 1.5 standard deviation of all differences between DNA and RNA parameters) in attraction towards nonpolar amino acids such as Val, Leu, Ala, and Trp. Second important difference between RNA and DNA – the absence of C2’ ribose hydroxyl group – also led to the expected changes in interaction parameters for GS2 bead: an increase in attraction towards hydrophobic Leu, Cys, and Trp, and a stronger repulsion towards hydrogen bond acceptors such as His or Asp. It should be noted, that the actual physical nature of interactions was not taken into account in our knowledge-based potential, and the observed changes in interaction preferences resulted solely from the selection pressure towards efficient scoring imposed in the force field optimization procedure.

Systematic docking searches were not able to find any hit for around 15% of structures (Table 2) from both training and test sets. In most of those cases (75%), the energy of best ranked solution was less favorable than the minimized energy of native complex. It suggests that more extensive sampling, for example by increasing the number of initial ligand placements and orientations for energy minimization, would allow finding some additional well scoring native-like structures and improve the results, similarly as it was observed in the case of protein-RNA docking [21].

### Comparison with experimental binding affinities

In principle, a reliable docking method should not only allow distinguishing between bound geometries and their corresponding decoys, but should also provide proper ranking of native complexes according to their absolute binding free energies. In order to test this ability, force field energy values obtained upon energy minimization of native complexes were compared with experimental affinities. Such data is available for a number of protein-DNA interactions [25], however, a meaningful association of thermodynamic parameters with relevant crystallographic structures is not a trivial task.

One needs to standardize multiple factors affecting experimental results such as: temperature, pH, ionic conditions, the presence of cofactors, or experimental method used. Furthermore, one should bear in mind that structures depicted by X-ray crystallography usually represent only fragments of macromolecules used for *in vitro* assays. Even, if a complete binding site is preserved, the influence of missing parts, such as flanking DNA segments, is impossible to estimate, albeit substantial in some cases. In an effort to minimize those effects, a strict selection process was introduced for matching structural and experimental data (see Methods). The already available dataset [44], used to date by several other groups, occurred to violate the adopted rules in most of the cases, nonetheless the results are provided for comparison.

**Figure 3:**
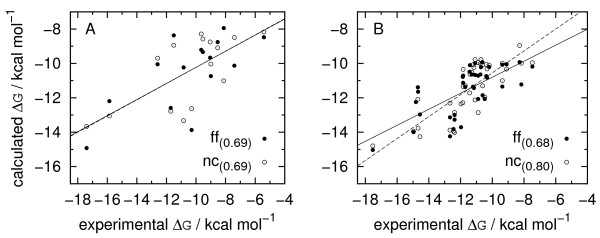
**Correlation between calculated and experimental binding free energies.A**: data set selected from ProNIT database, **B**: data set by Zhang *et al.*[44]. Filled circles – results for the full force field, empty circles – an estimate based on the number of native contacts.

The correlation (Pearson’s correlation measure is used here and in the following) between calculated and experimental binding free energies was at the level of 0.69 (Figure 3, A). This is certainly too low to gain quantitative insight into binding thermodynamics, and provides only a limiting accuracy level to distinguish between particularly strong and weak complexes. In addition, similar calculations with the use of non-specific force field (see Methods) resulted in correlation coefficient of 0.71. Furthermore, similar correlation (0.69) was obtained for the most crude estimate of binding free energy, based on the number of native contacts (receptor-ligand pseudoatom pairs closer than 8 Å to each other). Similar trend was observed for the alternative dataset (Figure 3, B), in which average sequence similarity between structures with known binding affinities and their geometric representations used in calculations was typically lower. Here, the correlation coefficient obtained for energy minimization in full protein-DNA force field was 0.68, and raised through 0.76 for minimization with non-specific parameter set, up to 0.80 for energy estimate based on native contacts.

Such results indicate that the correlation to experimental binding free energy is generally a poor measure of force field performance, in line with similar observations from other studies [23]. It may be understood, provided gross simplifications applied in computational approach. To name a few: all internal degrees of freedom, important for energetic and entropic effects due to conformational changes upon binding, are neglected along with the existence of solvent and ions, the available atomic coordinates in most cases represent only a part of the considered system, and finally, pairwise statistical potentials do not describe any physically sound free energy contributions [22]. Moreover, on the experimental side, affinity estimates for a given system provided by different sources can sometimes vary by few kcal/mol. In such circumstances, it seems reasonable that the interface size appears as the most meaningful estimate of binding free energy – after all binding strength should be on average proportional to the number of contacts between protein and DNA. The influence of “higher order corrections”, hopefully conveyed by force field details seems to be obscured by random effects of the aforementioned simplifications.

A more indicative estimate of the actual force field performance can be obtained by analyzing differences in affinities for a number of DNA mutants targeting the same protein partner. Here, one can assume limited variability of uncontrollable factors within each system of interest, and hence, the cancellation of their effect on relative free energies. A set of experimentally determined binding free energies with matching structural data was taken from the work of Morozov *at al.*[45]. It comprised 13 protein-DNA complexes, with a total of 293 *ΔG*measurements for different mutants (see Methods).

**Figure 4:**
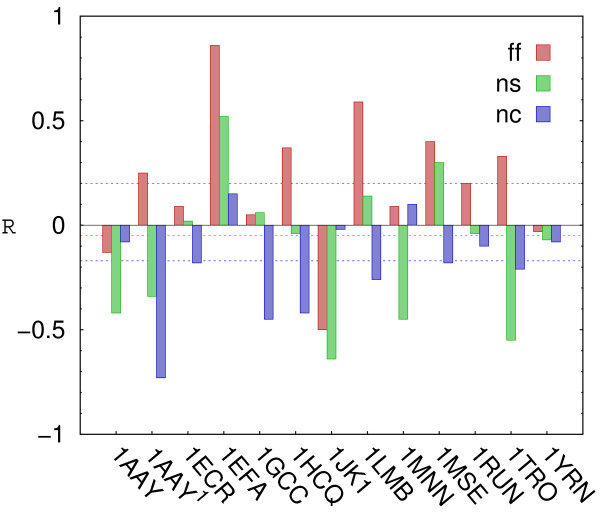
**Correlations between experimental and calculated binding affinities for: full protein-DNA (ff) and non-specific (ns) force fields, and an estimate based on the number of native contacts (ns).** Dotted lines correspond to weighted averages, with weights proportional to the number of experimental data points for each case.

An average correlation between experimental free energy differences and estimates obtained upon potential energy minimization in protein-DNA force field was 0.20 (Figure 4). This is certainly too low to claim that calculations can capture sequence specificity. It is important to note, however, that only rigid structures were considered for this test, thus no conformational changes in response to DNA mutations were allowed (indeed, including protein and DNA flexibility improved correlation – data not shown). Moreover, the differences in experimental binding free energies for closely related mutants were usually smaller than 0.5 kcal/mol, which is well below accuracy that can be reasonably expected for a coarse grained representation. Interestingly, only slightly better correlation (0.23) was observed in a similar test for the full atom Amber force field [23], indicating that achieving high sequence specificity is a formidable task even for more detailed models.

The non specific potential and contact-based estimate of interaction energy showed no predictive power in sequence specificity test, yielding average correlations with experimental data at levels of −0.05 and −0.17, respectively (Figure 4). It demonstrates that a positive correlation observed for the full protein-DNA potential, albeit small, was indeed due to sequence specificity. Apparently other factors, such as interface size or shape complementarity, had no predictive value. A negative correlation observed for contact-based predictions was most likely due to non-resolved sterical clashes being counted as favorable interactions. Energy minimizations were not performed in that case, and hence interfaces had adequate geometries only for DNA sequences found in crystallographic structures.

### Docking of unbound structures

Proper docking of macromolecules based on their unbound conformations requires the prediction of local and global structural rearrangements that likely occur upon binding. Several approaches have been adopted in order to tackle this problem, such as the use of multiple starting conformations [18], multiple copies of loops [46] or side chains [19], or the inclusion of global flexibility by considering low frequency normal modes [47]. All those methods are technically available within the framework of ATTRACT docking program [19,46,47]. Nonetheless, as the current report is focused predominantly on the development of interaction potential, only rigid body docking was considered to provide a baseline estimate of force field efficacy, not affected by additional factors.

A set of benchmark structures, containing 47 pairs of binding partners in their unbound conformations along with the corresponding assembled complexes, was taken from the work by van Dijk and Bonvin [48]. This set covers all major groups of protein-DNA complexes according to the classification of Luscombe at all [49], and provides cases with RMSD between bound and unbound conformations ranging from ∼1, up to ∼10 Å, both on protein and DNA side. The benchmark is non-redundant within itself in terms of sequence similarity, however, some structures (1RVA, 1K79, 1CMA, 1EMH, and 1KC6) are close to those already present in the training set. Due to relatively small size of the benchmark set, as well as the fact that the observed results for training and test sets were no different (Table 2, Figure 2), those structures were included into the study of unbound docking. The criteria used so far to define a ”hit” were extended to account also for “acceptable” solutions, that is having iRMSD ≤ 4.0 Å and *f*_*NC *_≤ 0.3, or *f*_*NC *_≥ 0.3, in line with CAPRI classification.

**Figure 5:**
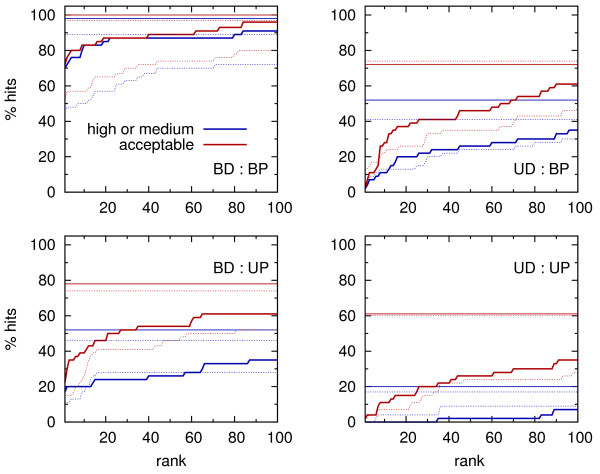
**Fractions of “high or medium”, and “acceptable” solutions among 100 top ranked docked geometries.** Dotted lines - results for non specific force field. Horizontal lines correspond to the respective fractions for all obtained geometries.

As a reference, bound-bound docking of all benchmark structures was also performed. “Hits” were found for almost all complexes (98%), and in 72% of the cases they corresponded to the best-ranked solutions (Figure 5). The statistics for “acceptable” solutions was only marginally better. This could be expected, provided that high or medium quality solutions were found in almost all cases, but just some of them were unfavorably scored – in such situation it was hardly possible that an alternative solution of only “acceptable” quality would achieve a better score.

**Figure 6:**
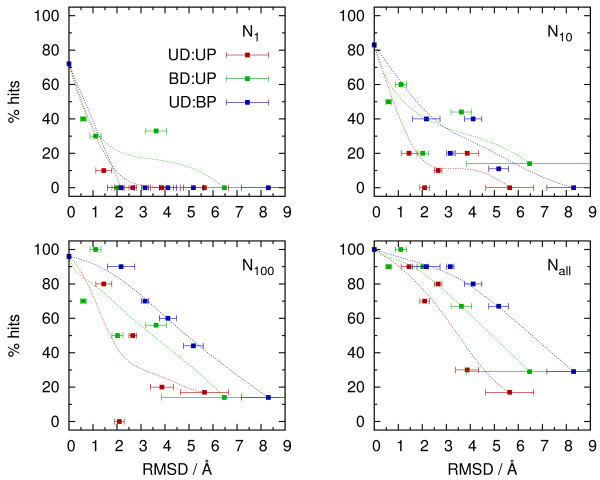
**Fractions of ”acceptable” solutions in: top ranked (*N***_**1**_**), 10 or 100 best ranking (*N***_**10**_, ***N***_**100**_**), and all docked geometries (*N***_***all***_**).** The results are presented as a function of average bound vs. unbound RMSD for sub-groups of the considered complexes, after sorting them according to the respective RMSD, and binning into groups of 10. Dotted lines (Béziere curves) are added for eye guidance.

In mixed bound-unbound docking, two scenarios were considered: unbound DNA - bound protein (UD:BP), and bound DNA - unbound protein (BD:UP). The total fractions of generally found “hits” and “acceptable” solutions were very similar in both cases: ∼50%, and ∼75% respectively (Figure 5). A closer inspection of the fraction of generally found “acceptable” solutions as a function of RMSD for the unbound component revealed, however, that at higher deformation levels significantly more native-like solutions were found in the UD:BP case (Figure 6, *N*_*all*_). On the contrary, scoring in this scenario was less efficient, with no top ranked “hits” nor “acceptable” solutions at all (Figure 5). To some extent, better scoring in BD:UP case was due to the fact that mildly deformed (with RMSD ∼1 Å), and hence easier to dock, structures were more abundant among unbound proteins (Figure 6, *N*_1_). Nonetheless, there was a group of complexes with unbound protein components having an average RMSD of 3.5 Å, and the fraction of top ranked “acceptable” solutions still around 30%, while *none* of UD:BP cases, with even lower RMSD for unbound DNA, had a top ranked native-like solution.

It indicates that structural distortions of the two binding components may have different effects on the docking process. Unbound DNA appears to be generally more effectively recognized by a protein binding partner preserved in its bound conformation, but scoring of the resulting complexes among the very best solutions (with rank up to 10) is less frequent. It may be a consequence of the fact that regular DNA secondary structure, with exposed phosphate backbone, may provide enough contacts to find a native-like binding mode, but its favorable scoring among other backbone-driven geometries may require some degree of sequence dependent deformation [10]. In such situations, all information necessary for specific recognition is lost when regular B-DNA is used for docking. On the unbound protein side, the lack of generic regular form, such as double helix, makes the ability to generally find a solution more dependent on the level of structural deviation from the bound geometry. At the same time, however, due to greater variability of protein building blocks, their unique constellation responsible for specific recognition is more likely to be preserved in unbound conformation, resulting in more efficient scoring for native-like BD:UP complexes.

The statistics for unbound - unbound docking was expectedly worse than for mixed cases. High or medium quality solutions were generally found for 20% of cases (“acceptable” solutions for 60%) and for only ∼10% of cases “hits” were among 100 top ranking geometries (“acceptable” solutions for 35% of cases). Interestingly, the overall performance with respect to high or medium quality solutions for the same unbound benchmark set was quite similar to the one of the HADDOCK docking program working in rigid docking mode. Two-star solutions (equivalent to “high or medium” quality used here) were generally found for 20% of cases, and roughly half of them (11% of the total number of cases) was scored preferably, that is with at least 10% two-star geometries among the considered best ranking 400 (please note that HADDOCK is based on a different docking approach and that different criteria were used for the evaluation of results). The reported results for “acceptable” solutions were somewhat better in the case of HADDOCK (76% of generally found and 60% of preferably scored solutions), however, it should be noted that an ensemble of 5 rigid protein geometries (obtained with the use of simulated annealing and refinement in explicit water) was submitted to docking for each complex, instead of a single, crystallographic geometry used here.

The analysis of results as a function of average RMSD for the two binding partners (Figure 6), indicates a fast initial drop in the number of well ranked solutions, followed by a plateau extending for RMSD between 2 and 4 Å, and further drop for larger RMSD values. This justifies the distinction of three groups of complexes with qualitatively different success rates, in line with designation of easy, intermediate and difficult cases in other studies [17,18].

A comparison with the results obtained for non-specific force field (Figure 5, dotted lines) underlines an important role of shape complementarity, at least for rigid body docking considered here. In case of bound - bound docking, shape complementarity alone seemed to be enough to provide favorable ranking in roughly two third of complexes. It’s role was greater in bound-unbound docking scenarios, and increased even further in unbound-unbound docking. At first sight it seems counterintuitive, as one would expect that the recognition of unbound cases should benefit more from specific interactions, in favor to geometric fit. It is important to note, however, that certain level of shape complementarity is required for specific contacts to occur. Apparently, docking of structures that need such specific contacts for proper ranking is particularly challenging, as first, a proper geometry needs to be found, and second, specific interactions need to be favorably accounted for by the force field. As a result, proper ranking of such cases is relatively less frequent in bound-unbound, or unbound-unbound rigid docking.

### Sequence-dependence in systematic docking

The results presented in previous sections bring into question the importance of direct sequence readout for the efficacy of systematic docking searches performed here. In order to test it, docking results for bound geometries were rescored at different levels of sequence identity to native DNA. In total, 41 complexes from the benchmark set, with “hit” found within top 10 docking solutions, were used for this task.

**Figure 7:**
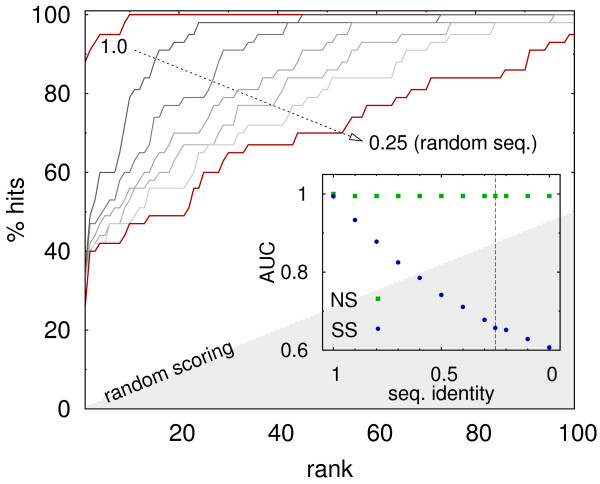
**Rescoring of systematic docking results for decreasing DNA sequence identity with respect to native structure.** Lines correspond to sequence identity of 1.0, 0.9,.0.5, 0.25, in order denoted by an arrow. “random scoring” level corresponds to a random distribution of “hits” within 200 scored geometries. Inset: “area under curve” (AUC) for sequence specific (SS) and non-specific (NS) complexes. Vertical line corresponds to random sequences (0.25 identity level).

The observed scoring efficacy apparently dropped with the increasing level of noise introduced to DNA sequences (Figure 7). “Hits” shifted towards lower ranks in around 50% of cases after being rescored with random DNA sequences. Still, however, in almost all cases (95%) they remained within 100 top solutions. The observed effect, although nonnegligible, indicates a limited role of direct sequence readout for scoring efficacy, at least with respect to an “average” complex from the analyzed pool.

One should bear in mind, however, that some protein-DNA complexes are naturally sequence non-specific. Indeed, at least 5 complexes (1AZP [50], 1QRV [51], 1DIZ [52], 1VAS [53], 4KTQ [54]), i.e. roughly 10% of the considered group, seem to belong to such category. Accordingly, ranking of their native-like geometries did not depend on DNA sequence (Figure 7, inset). Furthermore, the origin of sequence specificity among the remaining, “specific” complexes, likely involves a combination of direct and indirect (geometry dependent) readout mechanisms in varying proportions [10]. For example, at least 3 out of 15 complexes, that are regarded as “specific” but had their native-like geometries ranked among 10 top docking solutions for random DNA sequences, are known to form mainly due to indirect readout mechanism (these are: 1QNE [55], 1TRO [56], 1ZME [57]).

Unfortunately, no general correlation (with |*R*|>0.2) was found between the tendency of “hit” rank to remain unchanged in spite of sequence perturbation (which could indicate the domination of indirect readout mechanism) and descriptors such as deviation from ideal B-DNA conformation, the amount DNA backbone involved in complex formation, or interface size. Nonetheless, the observed “limited” role of direct sequence readout at least to some extent reflects the complex nature of protein-DNA recognition, and thus should not be attributed solely to force field deficiencies.

## Conclusions

A coarse grained force field for protein-DNA docking was presented. It is compatible with previously developed parameter sets for protein-protein and protein-RNA docking, all suitable to use within the ATTRACT docking protocol. The force field was parametrized in a knowledge-based manner on a set of available protein-DNA complexes. Its ability to reconstruct native complexes based on systematic docking of bound components was tested on an independent test set, yielding very good results. The quality of predictions in bound-unbound and unbound-unbound docking scenarios was expectedly worse, depending on the level of structural deformation of the binding partners. It is worth stressing, however, that the overall performance was similar to the one acheived by methods relying on the knowledge of the true interface, even though no such information was utilized here.

The energy score for docked complexes was shown to correlate with experimental binding free energies, however, the correlation could be likely explained solely by taking into account the number of protein-DNA contacts, without truly meaningful input from specific amino acid - nucleotide interactions. The role of such specific interactions was further investigated by comparing experimental and calculated effects of DNA mutations on binding free energy, as well as monitoring the sensitivity of docking results on random DNA sequence alterations. Both tests revealed certainly notable, yet relatively limited importance of sequence specific interactions both for the reproduction of experimental effects and favorable scoring of native sequences.

The extent to which it reflects true aspects of protein-DNA recognition, in which considerable effect is attributed to sequence specific DNA deformation and not only to specific intermolecular contacts, remains an open question. Certainly, however, the above findings pose a serious challenge to flexible protein-DNA docking algorithms. In order to avoid false positive shape complementarity the allowed conformational changes would have to remain strictly in physically sound regime, and furthermore, their associated free energy change would have to be adequately quantified and included into the scoring scheme. Addressing those challenges is within the scope of our current efforts.

## Competing interests

The authors declare that they have no competing interests.

## Author’s contributions

PS parametrized the force field, collected experimental data, carried out docking, interpreted the results, was involved in writing the manuscript, RB compiled the set of protein-DNA complexes, was involved in writing the manuscript, MZ designed the project, was involved in writing the manuscript. All authors read and approved the final manuscript.

## Supplementary Material

Additional file 1 **Supporting information.** Supporting information in pdf format contains the list of all protein-DNA complexes used for parametrization and testing. Click here for file
