# Supplementary material for: Protein-DNA docking with a coarse-grained force field
Source: BMC Bioinformatics. 2012 Sep 11;13:228. doi: 10.1186/1471-2105-13-228 (PMC3522568; doi:10.1186/1471-2105-13-228)
Supplement: Additional file 1 — Supporting information. Supporting information in pdf format contains the list of all protein-DNA complexes used for parametrization and testing. [file 1471-2105-13-228-S1.pdf]

Protein-DNA docking with a coarse-grained force field.

Supporting information.

Piotr Setny[1], Ranjit Bahadur[2], and Martin Zacharias[1]

[1]Physics Department T38, Technical University Munich, James Franck Str. 1, 85748 Garching, Germany

[2]Indian Institute of Technology Kharagpur, Kharagpur - 721302, India

Table 1. List of protein-DNA complexes.

Pdb id	Complex name	DNA chains	Protein chains	Resol-ution (Å)
1am9	HUMAN SREBP-1A BOUND TO LDL RECEPTOR PROMOTER	GF(17,21)	AB(82/82)	2.3
1au7	PIT-1 MUTANT/DNA	CD(25,24)	AB/CD(146/146)	2.3
1b3t	EBNA-1 NUCLEAR PROTEIN/DNA	CD(18,18)	AB(147,147)	2.2
1b94	RESTRICTION ENDONUCLEASE ECORV	CD(11,11)	AB(244/244)	1.9
1bdt	ARC/DNA	EF(22,22)	AB(53,53)	2.5
1bl0	MULTIPLE ANTIBIOTIC RESISTANCE PROTEIN (MARA)/DNA	BC(24,24)	A(129)	2.3
1cw0	VERY SHORT PATCH REPAIR (VSR) ENDONUCLEASE WITH A DUPLEX DNA	MNO(12,3,9)	A(155)	2.3
1d2i	RESTRICTION ENDONUCLEASE BGLII WITH 16-MER DNA2	CD(16,16)	AB(223,223)	1.7
1d3u	TRANSCRIPTION FACTOR (II)B/BRE+TATA BOX	CD(24,23)	AB(181,201)	2.4
1dc1	RESTRICTION ENZYME BSOBI/DNA	WC(13,13)	AB(323,323)	1.7
1dtz	CAMEL APO-LACTOFERRIN	EF(13,13)	A(282)	2.65
1ebm	8-OXOGUANINE GLYCOSYLASE (HOGG1) BOUND TO A SUBSTRATE OLIGONUCLEOTIDE	CD(15,15)	A(317)	2.1
1eyu	PVUII ENDONCULEASE/COGNATE DNA	CD(13,13)	AB(157,157)	1.78
1f44	CRE RECOMBINASE-LOX COMPLEX	MN(16,19)	A(324)	2.05
1f4k	REPLICATION TERMINATOR PROTEIN/B- SITE DNA COMPLEX	DE(121,121)	AB(122,122)	2.5
1f6o	AAG DNA REPAIR GLYCOSYLASE COMPLEXED WITH DNA	DE(13,13)	A(129)	2.4
1gxp	PHOB EFFECTOR DOMAIN IN COMPLEX WITH PHO BOX DNA.	CD(23,23)	AB(106,106)	2.5
1h6f	HUMAN TBX3, BOUND TO A PALINDROMIC DNA	CD(24,24)	AB(193,193)	1.7
1h89	TERNARY PROTEIN-DNA COMPLEX2	DE(26,26)	ABC(64,64,159)	2.45
1hcr	HIN RECOMBINASE BOUND TO DNA	BC14,13)	A(52)	2.3
1hlv	CENP-B(1-129) COMPLEXED WITH THE CENP-B BOX DNA	BC(21,21)	A(131)	2.5
1f3j	HISTOCOMPATIBILITY ANTIGEN I-AG7	BC(21,21)	A(116)	3.1
1iaw	NAEI COMPLEXED WITH 17MER DNA	CDEF(17,17,17,17)	AB(317,317)	2.4
1ign	DNA-BINDING DOMAIN OF RAP1 WITH TELOMERIC DNA	CD(19,19)	A(246)	2.25
1j1v	DNAA DOMAIN IV COMPLEXED WITH DNAABOX DNA	BC(13,13)	A(94)	2.1
1je8	NARL/DNA COMPLEX	CD(20,20)	AB(82,82)	2.12

1jj4	HUMAN PAPILLOMAVIRUS TYPE 18 E2 DNA-BINDING DOMAIN BOUND TO ITS DNA	CD(16,16)	AB(83,83)	2.4
1k4t	HUMAN DNA TOPOISOMERASE I (WITH THE POISON TOPOTECAN AND COVALENT COMPLEX WITH A 22 BASE PAIR3 DNA DUPLEX	BCD(10,12,22)	A(592)	2.1
1k78	PAX5(1-149)+ETS-1(331-440)+DNA	GH(27,27)	EFI(149,110,149)	2.25
1ku7	RNA POLYMERASE SIGMAA SUBUNIT REGION 4 BOUND TO-35 ELEMENT DNA	BC(11,11)	A(73)	2.4
1l3l	QUORUM-SENSING TRANSCRIPTION FACTOR COMPLEXED WITH PHEROMONE AND DNA	FH(20,20)	AC(234,234)	1.66
1le8	MATA1/MATALPHA2-3A HETERODIMER BOUND TO DNA COMPLEX	CD(20,20)	AB(53,83)	2.3
1lq1	DNA COMPLEXED STRUCTURE OF THE KEY TRANSCRIPTION FACTOR INITIATING	GH(16,16)	AB(120,120)	2.3
1mj2	METHIONINE REPRESSOR MUTANT (Q44K) PLUS COREPRESSOR (S- ADENOSYL METHIONINE) COMPLEXED TO A CONSENSUS OPERATOR SEQUENCE	FG(19,19)	ABCD(104,104,104,104)	2.4
1mus	TN5 TRANSPOSASE/DNA	BC(20,20)	A(477)	1.9
1nfk	NF-KB P50	CD(11,11)	AB(325,325)	2.3
1nh2	TFIIA/TBP/DNA COMPLEX	EF(16,16)	ACD(180,79,121)	1.9
1njz	CYTOSINE-THYMINE MISMATCH AT THE POLYMERASE ACTIVE SITE	BC(10,16)	A(580)	2
1nkp	MYC-MAX RECOGNIZING DNA	FG(19.19)	AB(88,83)	1.8
1nlw	MAD-MAX RECOGNIZING DNA	FG(18,18)	AB(80,76)	2
1oe4	SMUG1/DNA	EF(12,12)	AB(247,247)	2
1ozj	SMAD3-MH1 BOUND TO DNA	CD(15,15)	AB(144,144)	2.4
1pm5	FPG/DNA	DE(14,14)	A(271)	1.95
1pp7	INITIATOR BINDING PROTEIN BOUND TO THE FERREDOXIN INR	EF(13,13)	U(131)	2.45
1pt3	NUCLEASE-COLE7/OCTAMER DNA	CDGH(8,8,8,8)	A(128)	2.5
1puf	HOXA9 AND PBX1 HOMEODOMAINS BOUND TO DNA	DE(20,20)	AB(77,73)	1.9
1qum	ENDONUCLEASE IV / DAMAGED DNA	BCD(6,7,13)	A(285)	1.55
1r7m	HOMING ENDONUCLEASE I-SCEI/DNA	CD(25,25)	A(235)	2.25
1rh6	EXCISIONASE (XIS)-DNA COMPLEX	CD(15,15)	A/B(55,55)	1.7
1rio	CI-NTD/DNA	UT(27,27)	ABH(98,98,73)	2.3
1rm1	TFIIA/TBP/TATA-BOX DNA COMPLEX	DE(18,18)	ABC(240,122,286)	2.5
1skn	SKN-1 / DNA	AB(15,15)	P(92)	2.5
1ssp	URACIL-DNA GLYCOSYLASE BOUND TO URACIL-CONTAININGDNA	AB(10,11)	E(223)	1.9
1sxq	BGT/13MER DNA	CE(13,13)	A(351)	1.8
1tc3	TRANSPOSASE TC3A1-65/DNA	AB(21,20)	C(51)	2.45
1tkd	T7 DNA POLYMERASE/DNA	PT(22,26)	AB(698,108)	2.49
1tro	TRP REPRESSOR/DNA	IJ(19,19)	AC(108,108)	1.9

1u8b	METHYLATED N-ADA/DNA COMPLEX	BCDE(6,5,12,13)	A(113)	2.1
1vrl	MUTY ADENINE GLYCOSYLASE/ DNA	BC(11,11)	A(369)	2.5
1w0u	HTRF2 / TELOMERIC DNA.	CD(17,17)	AB(55,55)	1.8
1w7a	ATP BOUND MUTS	EF(30,30)	AB(800,800)	2.27
1wd0	CHROMOSOMAL PROTEIN SAC7D /DNA DECAMERS	BC(10,10)	A(66)	1.9
1zs4	CII / DNA	UT(27,27)	ABCD(83,83,83,83)	1.7
2ady	P53 TETRAMERS (COMPLEX IV) /DNA	EF(12,12)	AB(200,200)	2.5
2alz	HPOLI/DNA /DCTP	TP(9,7)	A(390)	2.5
2aor	MUTH HEMIMETHYLATED/DNA	CD(22.22)	A(223)	2
2aq4	REV1 / DNA/DCTP.	PT(12,16)	A(434)	2.32
2bam	RESTRICTION ENDONUCLEASE BAMHI / DNA	CD(12,12)	AB(213,213)	2
2bnw	RIBBON-HELIX- HELIX OMEGA REPRESSOR / DNA HEPTAD REPEATS	EF(18,18)	AB(53,53)	2.45
2c6y	INTERLEUKIN ENHANCER-BINDING / DNA	CD(16,16)	AB(111,111)	2.4
2c7o	HHAI DNA METHYLTRANSFERASE / 13MER OLIGONUCLEOTIDE	CD(13,13)	A(327)	1.9
2c9l	EPSTEIN-BARR VIRUS ZEBRA PROTEIN	AB(19,18)	YZ(63.63)	2.25
2cgp	CATABOLITE GENE ACTIVATOR PROTEIN/DNA	BC(11,15)	A(210)	2.2
2d5v	HNF-6ALPHA DNA-BINDING DOMAIN / TTR PROMOTER	CD(14,14)	A(164)	2
2ddg	URACIL-DNA GLYCOSYLASE / AP:G 2 CONTAINING DNA	CD(15,14)	A(219)	2.1
2dnj	DNASE I-OCTAMER	BC(8,6)	A(260)	2
2dtu	RB69 GP43 / DNA	EF(18,15)	A(896)	2.37
2etw	NDT80-MSE / DNA	BC(14,14)	A(345)	1.67
2ex5	HOMING ENDONUCLEASE I-CEUI / DNA	XY(26,26)	AB(207,207)	2.2
2fjx	RT29 / D(CTTGAATGCATTCAAG)	BG(8,8)	A(255)	1.8
2fkc	RESTRICTION ENDONUCLEASE HINP1I / DNA	CD(19,10)	A(247)	2.39
2g1p	DNA ADENINE METHYLTRANSFERASE (DAM) /DNA	FG(12,12)	AB(278,278)	1.89
2gih	Q138F HINCII / DNA GTCGAC	EF(14,14)	AB(257,257)	2.5
2h27	SIGMAE REGION 4 /-35 ELEMENT DNA	BC(12,12)	A(73)	2.3
2h7g	VARIOLA TOPOISOMERASE /DNA	YZ(12,14)	X(314)	1.9
2h7h	JUN BZIP HOMODIMER / AP-1 DNA	XY(19,19)	AB(62,62)	2.3
2hap	HAP1-18/DNA	AB(20,20)	CD(81,81)	2.5
2hos	HOMEODOMAIN / DNA	CD(21,21)	AB(63,63)	1.9
2hvr	T4 RNA LIGASE 2 /DNA	CDE(24,12,13)	A(335)	2.45
2i06	REPLICATION TERMINATOR PROTEIN (TUS) / DNA	BC(16,16)	A(309)	2.2
2i13	AART, A SIX FINGER ZINC FINGER /ANN TRIPLETS	CD(22,22)	A(190)	1.96
2ihm	MU /GAPPED 11MER DNA	TPD(11,6,4)	A(360)	2.4

2iie	SINGLE CHAIN INTEGRATION HOST FACTOR PROTEIN (SCIHF2) / DNA	CDE(35,15,20)	A(204)	2.41
2is6	UVRD/DNA/ADPMGF3	CD(25,25)	AB(680,680)	2.2
2isz	IDER-DNA	EF(33,33)	AB(157,157)	2.4
2jg3	MTAQI /BAZ	BC(10,10)	A(421)	1.9
2nll	THYROID HORMONE RECEPTOR DNA-BINDING DOMAIN /THYROID RESPONSE ELEMENT DNA	CD(18,18)	AB(66,103)	1.9
2oaa	RESTRICTION ENDONUCLEASE MVAI-/DNA	CD(11,11)	A(249)	1.5
2ofi	3-METHYLADENINE DNA GLYCOSYLASE I (TAG) BOUND TO 2 DNA/3MA	CB(12,12)	A(184)	1.85
2opf	DNA REPAIR ENZYME ENDONUCLEASE-VIII (NEI)2 / AP-SITE CONTAINING DNA SUBSTRATE	BC(12,12)	A(262)	1.85
2owo	DNA LIGASE / NICKED DNA ADENYLATE	BCD(26,13,13)	A(671)	2.3
2p0j	RESTRICTION ENDONUCLEASE BSTYI / NON-COGNATE DNA	CD(11,11)	AB(203,203)	2.1
2pi0	IRF-3 / PRDIII-I REGULATORY ELEMENT	EF(32,32)	AB(116,116)	2.31
2pyj	PHI29 DNA POLYMERASE /PRIMER-TEMPLATE DNA	XY(10,14)	A(575)	2.03
2q10	RESTRICTION ENDONUCLEASE BCNI (WILD TYPE)/ DNA	CD(11,11)	A(238)	1.75
2q2t	CHLORELLA VIRUS DNA LIGASE-ADENYLATE BOUND TOA 5' PHOSPHORYLATED NICK	BCD(21,10,11)	A(319)	2.3
2qkb	RNASE H /20-MER RNA/DNA HYBRID	CD(20,20)	AB(154,154)	2.4
2r1j	P22 C2 REPRESSOR PROTEIN /SYNTHETIC OPERATOR 9T	AB(20,20)	LR(68,68)	1.53
2vla	RESTRICTION ENDONUCLEASE BPUJI /DNA	LM(12,12)	A(285)	1.3
2yvh	TRANSCRIPTIONAL REPRESSOR CGMR /DNA	EFGH(14,14,14,14)	ABCD(177,177,177,177)	2.5
3bep	SLIDING CLAMP / DNA	CD(10,14)	AB(366,366)	1.92
3bs1	AGRA LYTTR DOMAIN / DNA	BC(16,16)	A(103)	1.6
3btx	ABH2 / DSDNA	BC(13,13)	A(204)	2
3c25	NOTI RESTRICTION ENDONUCLEASE / DNA	CD(22,22)	AB(383,383)	2.5
3c2i	MECP2 / METHYLATED DNA SEQUENCE FROM BDNF	BC(20,20)	A(97)	2.5
3clz	THE SET AND RING ASSOCIATED (SRA) DOMAIN OF UHRF1 /METHYLATED DNA	EF(12,12)	A(212)	2.2
3cro	THE PHAGE 434 CRO/OR1	AB(20,20)	LR(71,71)	2.5
6pax	PAX-6 PAIRED DOMAIN/DNA	BC(26,26)	A(133)	2.5
